# Advanced Life and Its Diseases

**Published:** 1909-02-20

**Authors:** R. Douglas Powell

**Affiliations:** Consulting Physician to the Middlesex Hospital, and Emeritus Lecturer in Medicine to the Medical School


### Hospital Clinics.

### ADVANCED LIFE AND ITS DISEASES.

By Sir R. DOUGLAS POWELL, Bart., K.C.V.O., M.D., F.R.C.P.;
Consulting Physician to the Middlesex Hospital, and Emeritus Lecturer in Medicine to the Medical School.

(Being the second of three lectures delivered at the Middlesex Hospital, January 29, 1909.)*

Gentlemen,—There are two or three points which I desired to make in my last lecture, namely, first, that old age is not in itself a disease, but an orderly change. Secondly, that many of the so-called diseases of old age, such as marked arterial sclerosis, or prostatic disease, are diseases which, for the most part, originate in the decade, from 55 to 65, preceding what arbitrarily we speak of as the commencement of old age. It is, however, only those who escape these pathological changes in the preceding decade or towards the end of it who really are, so to speak, candidates for longevity. Thirdly, that although grave arterial changes are not of the essence of old age, it is uncommon for people to enter upon that period without some degree of atheroma of vessels, which may therefore be spoken of as normal to this time of life. It is only when the changes are so out of proportion to the other changes of advancing senility that they become "disharmonies" and therefore disease.

Before dealing further with the disturbances of brain functions in old people, I should like to complete the little else which I have to say about the circulatory organs. Last year, in dealing with *angina pectoris*, including syncopal angina and the degenerations that lead up to it, I of necessity said a great deal about senile changes in the heart; and I need only very briefly touch upon that subject again at the present time. You will find most old people to have rather large hearts, and this condition may be said to be normal to them. In robust old people this is due to moderate hypertrophy, with slight, if any, dilatation. In others the heart is somewhat dilated and thin-walled. The natural vigour and habits of the individual have much to do with the condition of the heart. With daily exercise, and the vigorous respiratory and cardiac function which it entails, the coronary circulation is well maintained and the cardiac nutrition is well preserved. With a sedentary life the cardiac nutrition becomes languid. In cases, again, in which winter bronchial attacks are frequent there is an additional disposition to dilatation of the cardiac cavities, especially on the right side.

Apart from all these considerations, however, the integrity of the coronary arteries has to be reckoned with. If the aorta be atheromatous and the thickenings encroach upon the calibre of the coronary arteries, or if these latter be themselves atheromatous, the muscle of the heart withers, and is more or less replaced by fatty and fibrous tissues, and anginal and syncopal seizures are apt to occur. I need not further specify the pathology of the senile heart. It may be an organ quite adequate to carry on the functions of the old man, partaking only in his general decline. It may be even an unduly vigorous organ for his purpose; such an organ as that which the old gentleman who took a bath daily in the Serpentine must have enjoyed. It may be a thin-walled heart, dilated and weak from fibroid and other degenerations.

In old people with relatively weak and dilated hearts it is especially needful to take precautions against overloading the stomach. A large proportion of the cases which you will meet with of sudden heart failure in old people with weak hearts arise from their taking a large meal and getting acute dyspepsia, with flatulent distension. For these people quietude should be particularly enjoined before and after meals. But between meals a little walking exercise adapted to their strength, and drives may be advised. It is very useful to advise old people to get into the habit of taking occasional full inspirations so as to stimulate the coronary circulation. And sometimes you will also find that as people get into advanced life there is great benefit from the employment of oxygen inhalations from time to time, perhaps two or three times a day. Small doses of strychnia are valuable, and the necessity for digitalis or strophanthus or convallaria may be judged from the pulse. I will only say that in prescribing strophanthus and convallaria it is well to make sure that the preparation has been standardised, comes from a good source, and is reliable. With regard to these drugs also I would lay great emphasis in the treatment of old people, upon their being used in very small doses, only sufficient to restrain irregularity and undue frequency of the pulse, and to give tone to the muscle of the heart.

It will, in these days of modern surgery, come with increasing frequency in your experience to have to decide whether an old person is able to take an anæsthetic with safety. Perhaps the most common occasion on which this question will come before you will be that of the old man with advanced prostatic disease who has, it may be, for some years been

* For the substance of the third lecture see "Senile Respiratory Disorders," The Hospital, December 7, 1907.

leading a catheter life. I have been concerned in many such cases, in which an opinion is sought as to whether it is safe for them to take an anæsthetic, and my conclusion invariably is that if the reasons for the operation are on other grounds sufficient the risk of the anæsthetic should be faced. This risk is indeed comparatively a small item in the account, provided always that it be administered by a skilled anæsthetist whose undivided attention is given to the patient. With regard to the choice of the anæsthetic, if you have any voice in it —and I think you ought to have—I would strongly urge you to advise chloroform rather than ether. And I say this because there is still a prejudice that ether is a cardiac stimulant, as it undoubtedly is under certain circumstances. But as an anæsthetic for old people, often with somewhat bronchitic and emphysematous lungs, it is very dangerous. The only case of a fatal result ensuing directly from the anæsthetic which I remember in an old man, really resulted from the use of ether, setting up bronchitis and causing asphyxia. Chloroform is far better. I take it the reason is that chloroform is much more easily eliminated than is ether, and is much less irritating to the lungs.

There is only one other condition in the heart of old people to which I need refer, and that is the frequent presence of a mitral regurgitation. The development of a mitral murmur in elderly people is very common, and is not necessarily of much importance. It may be due to stiffening of the valve or of the chordæ tendineæ, or it may be co-incident with moderate dilatation of the ventricle. If the first sound be not wholly replaced, and if the cardiac rhythm be fairly steady, and the pulse volume fairly good, you need not trouble about the existence of a murmur. Indeed, I have often expressed the view that many old people are the better for the existence of a certain degree of mitral reflux, since it neutralises the mechanism of acute over-distension of the ventricle, which is part of the phenomena of angina pectoris.

I will now resume my comments on the cerebral conditions of old people, which are so much associated with arterial changes and modifications of blood pressure. I spoke of transient aphasias due to anæmia of the territory of the middle cerebral artery consequent upon atheromatous changes in the vessel. If one of these cerebral vessels gets narrowed, then, with the same pumping force of the heart, its territory will be less vigorously supplied with blood; and if the heart becomes weakened by any temporary cause, such as fatigue, so as to reduce the blood pressure, it is obvious that this area will suffer more than the others, and hence there is a partial anæmia of the middle cerebral area. It is a condition of analogous mechanism to muscular cramp in the limbs, cardiac fatigue or failure, dependent upon obstructed blood supply to other departments. These transient aphasias are apt to follow mental excitement or fatigue. The cerebral area thus affected would recuperate less readily than the other areas, and hence would more easily suffer fatigue-failure. Rest and restoratives are the best treatment.

It requires caution to avoid hastily mistaking these attacks of anæmic aphasia for hæmorrhage or thrombosis. These attacks are very analogous, clinically, to the temporary aphasic attacks which are not uncommonly observed in association with migraine. I do not know whether physiologists now will allow us to regard the cerebral vessels as being supplied with vaso-motor nerves. I have always considered that temporary aphasias and disorders of speech in connection with migraine are due to anæmia from arterial spasm of the cerebral vessels in the areas affected:—(1) a partially obstructed vascular area in the presence of a lowered blood-pressure from any cause, or (2) a temporarily exhausted cerebral centre with obstructed blood supply will account for it.

I spoke last time of the importance of not only knowing your Medicine, but of learning your patient, and these are cases in which this observation is particularly appropriate. A certain degree of cerebral excitement occasionally is of great advantage to old people; it flushes their brains, which tend to become stagnant. I remember an old gentleman, over eighty, who would alarm all his family by getting into great passions over trivial things; and his chief trouble was the recollection of names. He had been a West Indian merchant, and had had a large acquaintance. Any failure to recall a name caused him to get much excited. He had a very clever nurse, and when the name was finally hit upon she made a note of it, and gradually collected a list of thousands of names, classified according to locality. She was thus often able to help him out of his difficulty, which, of course, made him very pleased. His daughters were in great terror about these outbursts; but I pointed out to them that the attacks were really salutary for the old man, and probably did him a great deal of good by flushing his brain and keeping him going for some time after. Of course, there was some danger that he might get apoplexy, but he did not.

Another old gentleman, an Oxford Professor, occasionally lapsed into a torpid and depressed condition. Then perhaps someone would ask him to give an address. And what with preparing the address and delivering it, he got quite lively for the next two or three days: the flushing of his brain did him good. Of course, in another case the fatigue following exercise-flushing of the brain would be attended with a good deal of depression; therefore I say you must learn your patient. Sometimes you find it useful to stimulate the brain a little by giving mild breathing exercises, such as will serve to strengthen the cardiac power and promote and sustain the general circulation. Sometimes you can get mild excitement by encouraging these old people to have conversations and interviews with their friends, provided they are not too prolonged.

I must not omit one point, which is not generally appreciated or recognised, and that is that in some cases—of which I have seen a few instances —the general arterial system, as may be judged by the softness of the pulse and the satisfactory condition of the heart, may be in perfect order, apparently some years younger than the patient, and yet there are unmistakable symptoms of defective cerebral circulation. I pointed out to you, in my lectures last year, that the same thing had been observed with regard to the coronary arteries of the heart, which are in some instances degenerated beyond those of the general arterial system. So there may be much atheroma in the cerebral arteries, whilst the vessels generally are free. These are, however, exceptional cases. One case I have in mind is that of a gentleman, over 80, who has, in his day, been of exceptional mental power and vigour, which he has never spared, and, one might say, he had worn his cerebral vessels beyond those of his general arterial system. But, again, I can quote another case, that of a lady, of almost exactly the same age, who has rather less than the average brain capacity, who has never used her brain, except in the efforts incident to an over-conscientiousness in carrying out the duties of a high position and a large family. Yet she is in exactly the same condition as the old man who had apparently specially worn his brain. You cannot account for these things: you have to accept the fact that in some people the brain vessels degenerate before the rest of the vessels of the body. It is however certainly true that more brains rust out than wear out.

It is scarcely necessary to point out that the condition of vessels that renders individual old people liable to transient aphasias, and similar affections of other cerebral areas, also tends to result in thrombosis or hæmorrhage. But these cerebral hæmorrhages and thromboses are in no sense special to advanced age, although they are of common occurrence. So I do not propose to refer to them further.

Loss of memory, and of emotional and motor control, which are often observed in old persons, are probably attributable to change in the cortical cells of the cerebrum and cerebellum; but these changes cannot be very great, and are rather a want of receptivity to passing events; for the memory for long-past events often remains good.

There is another curious feature of disturbed circulation—anæmia rather than hyperæmia, and so transient as not to suggest any permanent change, which may sometimes be observed in old people; and that is an illusory feeling as to their whereabouts. They are liable to phases of excitement or panic from a sense that they are away from home and cannot get back; and they will be restless, and even violent under these illusions. Such illusions, more frequently than not, occur during some passing illness.

Recollection of a case of this kind reminds me to mention the necessity, in dealing with the aged to prescribe small doses of drugs, especially two classes of drugs, one hypnotics and the other strychnia. Strychnine, most valuable in small doses for short periods in old people, is apt if too long continued or given with too heavy a hand to cause cerebral excitement, and to raise the arterial tension, producing restlessness. Two and a half minims of strychnia or nux vomica once or twice a day are usually quite enough. Similarly, veronal and sulphonal should be given in not more than five-grain doses to begin with; and very often two- or three-grain doses may be enough. I have seen the most profound jaw-dropped coma produced by an average dose of veronal in an old gentleman who was suffering from illusions with regard to his locality and was quite violent because he thought he was in some other house than his own. Chloretone, which is a synthetic product somewhere between acetone and chloroform—trichloracetobutylalcohol—valuable for sea-sickness, is also useful as a mild hypnotic, and it and bromural (a synthetic bromo valerianate of urea), in five-grain doses two or three times daily are much lighter and safer hypnotics, and will sometimes suffice to relieve the condition I have spoken of. Opium must also be used in very small doses, but when so used is often very valuable, two and a half drops once or twice repeated often having a very markedly beneficent effect. You may humour these old people by taking them imaginary journeys to another room and back. I am reminded to tell you of an anecdote, not exactly but somewhat to the point, in regard to our late friend the resident medical officer, Mr. Fardon, at this hospital. I had some years ago a very bad case of delirium tremens in an omnibus driver. He was taken downstairs to one of the special wards, and the poor fellow was, like most such cases, sleepless and particularly annoyed with one delusion, namely, that he was driving his horses, but they would not move, and he could not get them round the corner. He was "clicking" away and pulling at them and whacking them, sweating profusely with his exertions, and getting very excited. Mr. Fardon, taking in the situation, got a pair of reins and tied them to the end of the bed, and put the other ends into the man's hands, and the moment he handled them he got round the corner and went to sleep!

Another and more rare cerebral condition in old people, of which I have seen one or two examples, is the optical delusion or illusion of seeing patterns, or geometrical shapes, or books arranged in order, crowds of faces or flowers in cascades upwards and downwards. These will occur from time to time, for a few moments to a few hours together, or almost continuously sometimes for days together, even threatening serious hallucination. There is no constancy in these illusions, perhaps numbers of faces, most vividly portrayed, will appear, quite different faces, but natural-looking, resembling a crowd; and sometimes some of them appear distorted. In the case I have in mind none of them were known to the individual. The condition causes considerable distress, and is probably analogous to a variety of tinnitus aurium, also common in old people. One does not know the explanation, but one thinks of it as a kind of tinnitus or buzzing of the ophthalmic nerve instead of the auditory nerve. It is some altered circulatory condition, of a transitory kind, for intermissions are absolute, and the intervals may be of long duration. The attacks are not influenced by external conditions of light or darkness. They are perhaps most troublesome in the half-lights. Nor are they influenced by direct ophthalmic conditions, such as tension or cataract. Concentrating the attention on a definite object will dissipate these visions for a time, just as the aural buzzing will vanish while intensely listening to one particular sound. Bringing the hand up will dissipate the scene for the moment. In these conditions, like that of illusions as to locality, you must look to possible causes of excitement of the brain, such as emotional strychnia, alcohol, or that so valuable a stimulant for old people, oxygen. With regard to medicinal treatment, I have found the combination of hydrobromic acid and tincture of hop with spirit of chloroform most useful in such a prescription as this:—Ac. hydrobrom. dil. ♏xx., tinct. chlorof. ♏x., tinct. humuli, ʒss., in water with syrup of ginger, taken three times in the day or as an occasional dose. Dark-coloured spectacles have been found useful; wearing them in the room or when going out.

Whilst old people are immune from many specific diseases, they are very liable to septic affections of mucous membranes of the oral, gastro-intestinal, and bronchial tracts. They often become careless about the condition of the mouth, and the gums get into a septic condition, especially around the stumps of old teeth and from insufficient attention to tooth-plates. These things must be very carefully looked to, as many cases of senile dyspepsia, depressed general health, and even bacterial invasion of endocardial surfaces, which are broken by atheromatous changes, are traceable to oral sepsis in them. There are many pleasant and suitable antiseptic lotions for cleansing the mouth which I need not specify by name. A slight astringent in combination with the antiseptic is valuable, and I am in the habit of prescribing a lotion consisting of Hazeline, Glycerini boracis aa ℥ij., eau de Cologne ʒj., aquam rosæ ad ℥viij., one tablespoonful in a large wineglass of water as a mouth-wash and gargle. A soft badger's-hair tooth-brush, dipped into water, and then sprinkled with a little of the undiluted lotion should also be used to brush the gums. I daresay it may seem to you a little trivial to deal with these details, but you will find that many diseases in aged persons arise from insanitary conditions in the mouth. Indeed, in acute diseases in all people, one of the most important points to look out for and to instruct the nurse about is to keep the mouth cleansed. A patient with pneumonia or bronchitis is constantly expectorating material highly charged with bacteria, which if you allow it to stick about the mouth and gums and tongue it is very apt to favour a recurrence of the infection, besides setting up infective gastric catarrh.

The bacillus coli is particularly apt in old people to penetrate beyond the limits of its normal territory. Great irritability of the bladder, especially at night, in women particularly, is common, and it will be found in some cases that the urine contains the bacillus coli communis. This emphasises the necessity in them of keeping the bowel functions regular; and this they generally recognise, and they mostly have a favourite laxative for the purpose; it is unwise to prescribe afresh in such cases. Young practitioners sometimes forget to find this out. Mercurials are of great value in old people, making an easy motion for them, and are doubtless useful as antiseptics. As I said last time, I am in the habit of prescribing $\frac{1}{4}$ gr. of calomel and $\frac{3}{4}$ gr. blue pill with 1 gr. ext. hyoscyami to be taken with the customary laxative, whatever it may be. Such a pill should be taken once or twice a week, so as to improve the general condition. Reasonable doses of calomel often act as a tonic; whereas larger doses will depress them.

One has to beware of the frequency with which fæcal block occurs in the rectum of old people, especially old women, which is very readily overlooked, as it excites frequent actions of the bowels and tenesmus. A rectal examination will reveal the condition, which can be dealt with mechanically or by enemata. I have known some cases, particularly one, of fæcal block, in which such grave symptoms —loss of flesh, want of appetite, anæmia, and a particularly drawn look—have resulted that the case was mistaken for one of malignant disease. Yet by simply clearing the rectum of the fæcal mass in it the patient made a recovery and lived many more years. An occasional dose of castor oil will prevent the recurrence of the condition when it has once been recognised and removed.

With regard to the urinary condition, we have recently acquired, in urotropine and allied preparations, very valuable means for the disinfection of the contents of the bladder. The preparations of lactic acid ferment suggested by Metchnikoff are useful in attacking and controlling the bacterial flora in the large intestine. Metchnikoff pointed out that the ferment of lactic acid has a peculiar affinity for the bacillus coli, and tends to attack it. By administering, therefore, a certain amount of this lactic acid ferment—which is prescribed generally in tablets which should be given crushed and diffused in a little milk and water and sugar—before meals twice or thrice a day, a great deal of relief is given; it diminishes the abdominal distension and dyspepsia in old people. But, beyond this, attention to the bowels is necessary. One must not forget the susceptibility of the surfaces of the cardiac valves and aorta in old people, from degeneration of the endocardium, to bacterial invasion, and the slow fungating endocarditis occasionally to be met with from this cause; an additional reason for guarding against the incidence of oral or intestinal sepsis.

